# Machine Learning for Prediction of Cognitive Health in Adults Using Sociodemographic, Neighbourhood Environmental, and Lifestyle Factors

**DOI:** 10.3390/ijerph191710977

**Published:** 2022-09-02

**Authors:** Govinda R. Poudel, Anthony Barnett, Muhammad Akram, Erika Martino, Luke D. Knibbs, Kaarin J. Anstey, Jonathan E. Shaw, Ester Cerin

**Affiliations:** 1 Mary Mackillop Institute for Health Research, Australian Catholic University, Melbourne, VIC 3065, Australia; 2 Melbourne School of Population and Global Health, University of Melbourne, Melbourne, VIC 3010, Australia; 3 School of Public Health, The University of Sydney, Sydney, NSW 2006, Australia; 4 Public Health Unit, Sydney Local Health District, Camperdown, NSW 2050, Australia; 5 School of Psychology, University of New South Wales, Sydney, NSW 2052, Australia; 6 UNSW Ageing Futures Institute, University of New South Wales, Sydney, NSW 2052, Australia; 7 Neuroscience Research Australia, Sydney, NSW 2031, Australia; 8 Baker Heart and Diabetes Institute, Melbourne, VIC 3004, Australia

**Keywords:** physical activity, neighbourhood environment, sedentary behaviour, machine learning, built environment, processing speed, cognition, memory, sociodemographic, prediction

## Abstract

The environment we live in, and our lifestyle within this environment, can shape our cognitive health. We investigated whether sociodemographic, neighbourhood environment, and lifestyle variables can be used to predict cognitive health status in adults. Cross-sectional data from the AusDiab3 study, an Australian cohort study of adults (34–97 years) (*n* = 4141) was used. Cognitive function was measured using processing speed and memory tests, which were categorized into distinct classes using latent profile analysis. Sociodemographic variables, measures of the built and natural environment estimated using geographic information system data, and physical activity and sedentary behaviours were used as predictors. Machine learning was performed using gradient boosting machine, support vector machine, artificial neural network, and linear models. Sociodemographic variables predicted processing speed (*r*^2^ = 0.43) and memory (*r^2^* = 0.20) with good accuracy. Lifestyle factors also accurately predicted processing speed (*r^2^* = 0.29) but weakly predicted memory (*r^2^* = 0.10). Neighbourhood and built environment factors were weak predictors of cognitive function. Sociodemographic (AUC = 0.84) and lifestyle (AUC = 0.78) factors also accurately classified cognitive classes. Sociodemographic and lifestyle variables can predict cognitive function in adults. Machine learning tools are useful for population-level assessment of cognitive health status via readily available and easy-to-collect data.

## 1. Introduction

Cognitive decline in ageing populations is a leading cause of disability and dependency worldwide. Emerging research suggests that sociodemographic factors, physical and social features of the neighbourhood environment, and lifestyle behaviours can shape the trajectory of cognitive health [1,2,3]. Older age and lower socioeconomic status are associated with an increased risk of cognitive decline, whereas higher level of educational attainment decreases the risk [2,4]. Some aspects of the neighbourhood environment, such as walkability, access to public transport, and services and facilities for recreational activities may contribute to better cognitive function in adulthood by supporting a healthy and active lifestyle [1]. There is evidence that regular physical activity is important for maintaining cognitive health [5], with even moderate-intensity physical activity such as utilitarian walking and gardening helping to reduce the risk of cognitive decline. Despite growing awareness about the impacts of our environment and behaviour on population-level cognitive health, research on whether individual risk of cognitive decline can be predicted using these variables is limited.

Previous studies have used model-based analysis to disentangle the impact of environmental attributes on cognitive health [6]. Two recent studies employed generalised additive mixed models to demonstrate that urban environmental characteristics, such as percentage of commercial land in residential areas, can have both positive effects on cognitive functions such as working memory and processing speed by facilitating engagement in physical activity [1], as well as negative effects through associated higher levels of ambient air pollution [7]. Using multilevel logistic regression models, Wu et al. reported positive associations between the distance to various services and the risk of dementia [8]. Similarly, Clarke et al. [9] employed three-level growth curve models to examine the relationship between the presence of community centres and cognitive function trajectories and found that such facilities protected against cognitive decline. These and other model-based studies [10,11] have allowed researchers to develop and examine theoretical ecological models of cognitive health [12].

While the previous studies have been very useful for better understanding the relationship between environment and cognition at a population level, studies focused on identifying the factors predictive of individual profiles of cognitive health are limited. Supervised machine learning is an emerging analytical approach with utility for prediction models based on large-scale individual profile data sources. Such models allow simultaneous assessment of the predictive ability of easy-to-collect external factors on cognitive health at both a group and individual level [13,14]. Sociodemographic data such as age, gender, education, and socioeconomic status can be easily collected and are often available via digital data collection, such as electronic health records [14]. Neighbourhood environmental characteristics can be estimated using open-source geospatial databases [1,15]. The availability of lifestyle data, such as rates of physical activity, is becoming ubiquitous due to increased use of wearables and mobile apps that can record lifestyle behaviours. Therefore, there is growing interest in and ability to leverage such cost-effective data for predicting individuals’ risk of cognitive decline based on factors posited by ecological models of cognitive heath.

Recent studies have used machine learning to predict cognitive decline [4] and future incidence of Alzheimer’s disease [14] using population-level sociodemographic and health data. However, there is a significant gap in our understanding of models and factors that are suitable for predicting specific domains of cognitive functions in middle-aged and older adults. This study aimed to address this gap by comparing the performance of four different machine learning models using sociodemographic, neighbourhood environmental, and lifestyle variables to predict two specific domains of cognitive functions in processing speed and memory. To achieve this aim, we extracted composite measures of built environment complexity and natural environment measured using geographic information system data. We then used these measures along with self-reported sociodemographic, physical activity, and sedentary behaviours as features in machine-learning-based analysis in a large, population-representative sample of younger and older adults.

## 2. Materials and Methods

### 2.1. Data Source

Data for this study are from the third wave of the Australian Diabetes, Obesity and Lifestyle study collected in 2011–2012 (AusDiab3) [16,17]. AusDiab is a population-based study that collected baseline data in 1999–2000 on 11,247 adults aged 25 years and older. The participants were selected from 42 areas (contiguous census units) in metropolitan and regional cities across Australia. The response rate relevant to this study was 67%. The third wave included cognitive function data from participants who attended testing sites for biomedical examination (*n* = 4614). The retention rate from baseline was 44.6%; with 14.5% of loss of follow-up attributed to death. Four hundred and seventy-three participants who did not reside in urban areas (defined as towns and cities of 10,000 people or more) were excluded, as the study focus was on urban areas. Therefore, the final analytical sample used in this study consisted of 4141 adult (25 years and older) urban community dwellers across 1286 Statistical Areas Level 1 (SA1s) in Australia [1]. The participants had no physical or intellectual disabilities and were residing at their address for at least 6 months prior to the survey. Surveys and cognitive tests were administered in English and in person by interviewers. Detailed protocols for the AusDiab study have been published elsewhere [17]. This study was conducted according to the guidelines of the Declaration of Helsinki, and approved by the Alfred Hospital Ethics Committee, Melbourne, Australia (ref. no 39/11; 2 March 2011).

### 2.2. Predictors

#### 2.2.1. Sociodemographic Predictors

The sociodemographic variables included in the study were: age, sex, educational attainment, household income, living arrangements (living with partner and no children; living with partner and children; living alone; other living arrangements), ethnicity (English-speaking vs. non-English-speaking background), and the index of relative socioeconomic advantage and disadvantage (IRSAD). All sociodemographic data except IRSAD were self-reported by the participants.

#### 2.2.2. Built and Natural Environment Predictors

Environmental variables quantified different aspects of the participants’ neighbourhood built and natural environments and were derived using ArcGIS v.10.5 software (ESRI, Redlands, CA, USA). A participant’s neighbourhood was defined as a 1 km-radius area surrounding her/his geocoded residential address [1,18]. The built environmental measures included population density (persons/ha), residential dwelling density, street intersection density (intersections/km^2^), percentage of commercial land use, percentage of residential land use, road-line density for major roads, road-line density for minor roads, and aerial distance to the nearest train line.

Natural environmental measures were percentage of parkland, percentage of blue spaces (e.g., lakes, coastlines, rivers, and reservoirs), aerial distance to parkland, and aerial distance to blue space. The sources of data used for computing these variables included the Australian Bureau of Statistics (ABS) Mesh Block data from the 2011 Census [19], the PSMA Australia’s 2012 Transport & Topography dataset [20], and the national topographic spatial data for surface water features sourced from Geoscience Australia [21].

Two ambient air pollutants were also included as predictors. They were nitrogen dioxide (NO_2_, units: ppb) and fine particulate matter <2.5 μm in aerodynamic diameter (PM_2.5_, units: μg/m^3^). Both pollutant exposures were estimated at each residential address using satellite-based land-use regression models, with the development and validation of the models described in detail elsewhere [22,23,24].

#### 2.2.3. Lifestyle Predictors

The lifestyle variables used in the study are self-reported measures of physical activity and sedentary behaviours. The physical activity measures included previous-week frequencies of engagement in transportation walking, leisure-time walking, vigorous gardening, and resistance training. The measures of sedentary behaviour included previous-week average daily hours of sitting time for transport, sitting time for leisure (including TV time), non-work computer-screen sitting time, sitting time for work, and sitting time for other purposes. Further details on these measures are reported elsewhere [1].

### 2.3. Outcome Measures

The participants were tested with three cognitive tests including the California Verbal Learning Test (CVLT) [25], the Symbol–Digit Modalities Test (SDMT), and the Spot-the-Word (STW) test. The STW test was not used in the current study as it is a measure of premorbid intelligence rather than of a current cognitive function. The CVLT is a measure of memory function [25]. In the CVLT test, 16 common shopping list items were presented to the participants. The score on the test was determined by the number of items correctly recalled after a 20 min delay following the presentation of the items. The SDMT is a measure of processing speed function. Processing speed is a key measure of executive function that is linked to healthy communication within brain networks [26]. In the SDMT test, participants used a reference key to find and orally report the numbers (1 to 9) corresponding to 9 geometric figures as quickly as possible. The score represented the number of correct responses given in 90 s.

Cognitive outcome measures were operationalised in two ways: (1) as continuous variables (raw SDMT and CVLT scores), and (2) as categorical variables (membership to latent classes representing profiles of SDMT and CVLT scores). Latent profile analysis of two cognitive function variables were used to identify latent classes. Models were estimated by the Expectation Maximization algorithm initialized by hierarchical model-based agglomerative clustering. The best-fitting model was selected based on the model that achieved the lowest Bayesian information criterion values. Once the best model was selected, participants’ class membership (i.e., membership to a specific cognitive function profile) was determined by the highest probability of class membership. 

### 2.4. Model Fitting and Evaluation

The model fitting and evaluation was performed in ‘R’ [27]. The analysis steps included: (1) multiple imputations into 10 datasets to account for the missing data, (2) latent profile analysis to identify any latent classes present in the cognition function data, (3) machine learning-based regression and classification analysis, and (4) pooling of performance measures across the 10 imputations. The source code developed for the model fitting and evaluation has been made openly available at github.com/govin2000/coghealth.

#### 2.4.1. Multiple Imputations

To account for missing data, 10 imputed datasets were created for the machine learning analyses. Multiple imputations by chained equations were performed using the package ‘mice’ in ‘R’ [1].

#### 2.4.2. Machine Learning Models

For the machine learning-based modelling, we used support vector machine (SVM), artificial neural network (ANN), gradient boosting machine (GBM), and regression models (linear models/logistic regression) to predict cognitive health. These four models represent different families of machine learning models, encompassing both information-based and error-based learning. We used separate regression (on continuous variables) and classification-based (on latent classes) approaches to compare the performance of models. For each machine learning model, seven different combinations of predictors were used for training and testing. This included sociodemographic (SDF), neighbourhood environment (NEF), and lifestyle factors (LSF), and their combinations (i.e., SDF + NEF, SDF + LSF, NEF + LSF, and SDF + NEF + LSF).

#### 2.4.3. Training and Testing Data

The dataset was randomly partitioned into training and testing sets using the well-established Pareto principle (80–20 split). The training set contained 80% of the observations that were used for model selection and tuning. Ten-fold cross validation was used for cross-validation during training. The best combination of hyperparameters for the machine learning models (for GBM and ANN) were chosen using grid search during cross-validation. The testing set contained the other 20% of the observations that were fitted using the trained models. The testing set results were used for model performance evaluation.

#### 2.4.4. Machine Learning-Based Regression

In the machine learning analysis for predictive continuous outcomes, the individual scores on both memory and processing speed were used as outcome measures. Root mean squared error (RMSE) was used as an error metric for model tuning and optimisation during cross-validation. The trained and tuned model was then used to evaluate performance on the separate testing set, which predicted individual memory and processing speed scores. The prediction accuracy on the test set was measured using a goodness of fit measure (coefficient of determination: R-squared error) between predicted and measured cognitive scores. The machine learning models were run for all 10 imputations of the data. The estimates of prediction accuracy and 95% CI were generated by applying Rubin’s rule [28] on Fisher’s Z-transformed R-squared values (*r^2^*). As per the previous guidelines, the *r^2^* from 0.02 to 0.12 was considered weak, 0.13 to 0.25 moderate, and 0.26 and above was considered substantial [29].

#### 2.4.5. Machine Learning-Based Classification

For machine learning classification, the cognitive classes identified using latent profile analysis were used as categorical outcomes for the machine learning analysis. Area under the receiver operating characteristic curve (AUC) was employed as a metric of classification accuracy during model cross-validation and tuning. The testing dataset was used to evaluate the performance of each machine learning model (and combination of predictors). Classification accuracy on the test dataset was determined using AUC. The machine learning models were run for all 10 imputations of the data. The estimates of classification accuracy (AUC) and 95% CI were generated using Rubin’s rule [28].

## 3. Results

### 3.1. Sample Characteristics

Table 1 shows the sociodemographic, neighbourhood environmental, and lifestyle and cognitive characteristics of the study participants. The mean age of the sample was 61 years, with a range of 34 to 97 years. Most participants were of English-speaking background, in paid employment, and with post-secondary education. The distributions of sex and household income were relatively balanced.

### 3.2. Machine Learning-Based Regression

The goodness of fit between predicted and measured processing speed (SDMT) and memory scores (CVLT) is shown in Table 2. Both sociodemographic [*r^2^* = 0.43, 95% CI = 0.37, 0.49, GBM and LM models] and lifestyle factors [*r^2^* = 0.29, 95% CI = 0.23, 0.35, GBM model] were good predictors of processing speed. However, neighbourhood environmental factors were weak predictors (*r^2^* < 0.05) across all models. When the sociodemographic and lifestyle factors were combined, the model performance only improved slightly [*r^2^* = 0.46, 95% CI = 0.41, 0.52, GBM model]. Model performance did not improve by further addition of the neighbourhood environmental factors.

The sociodemographic factor [*r^2^* = 0.20, 95% CI = 0.14, 0.27] was a moderate predictor of memory across all models. However, both neighbourhood and lifestyle factors were weak predictors of memory across all models (*r^2^* < 0.11). The combination of sociodemographic variables, neighbourhood environment, and lifestyle factors only slightly improved the model performance (Table 2).

### 3.3. Machine Learning-Based Classification

#### 3.3.1. Latent Profile Analysis—Defining Classes of Cognitive Function Profiles

Latent profile analysis was used to define classes of cognitive function profiles using the processing speed and memory scores. The 2-class model with ellipsoidal distribution, equal shape and volume, and variable orientation (EEV) was shown to have the lowest BIC value (Supplementary Figure S1). Therefore, the data were indicative of the 2-class model (high and low) best fitting the population.

#### 3.3.2. Classification Performance

**Figure 1:**
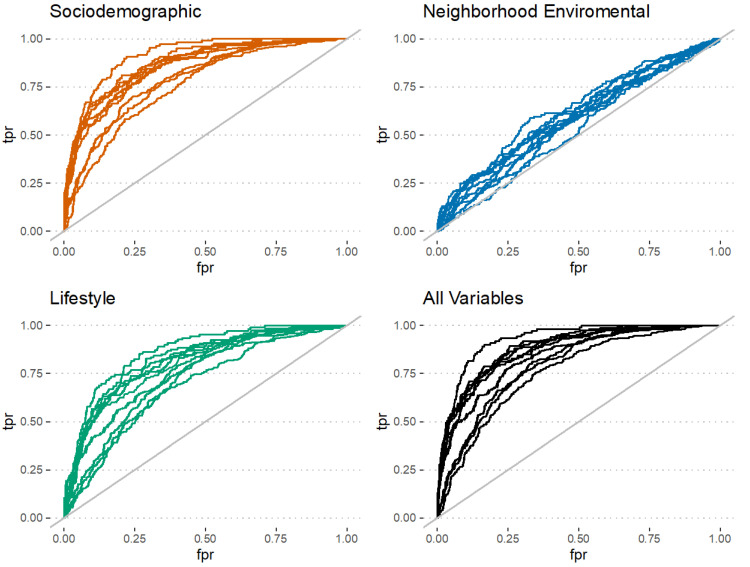
Performance of gradient boosting machine (GBM) model. Receiver operator characteristics (ROC) curves for prediction of cognitive classes using sociodemographic, neighbourhood environment, and lifestyle variables and their combination via GBM model. Ten separate ROC curves are shown for the 10 multiple imputations of the data. tpr = true positive rate, fpr = false positive rate.

AUC values for the classification models trained to predict the latent cognitive function classes are shown in Table 3. Sociodemographic factors [AUC: 0.85, 95% CI: (0.67, 0.94), LM] were good predictors when using all models. Lifestyle factors [AUC: 0.78, 95% CI: (0.61, 0.89)] were also good predictors when using the GBM model. Neighbourhood environmental factors were poor predictors (<0.7) of cognitive function classes. The prediction was not substantially improved when variables were combined. ROC curves for different combinations of variables are provided in Figure 1.

### 3.4. Relative Influence of Variables

**Figure 2:**
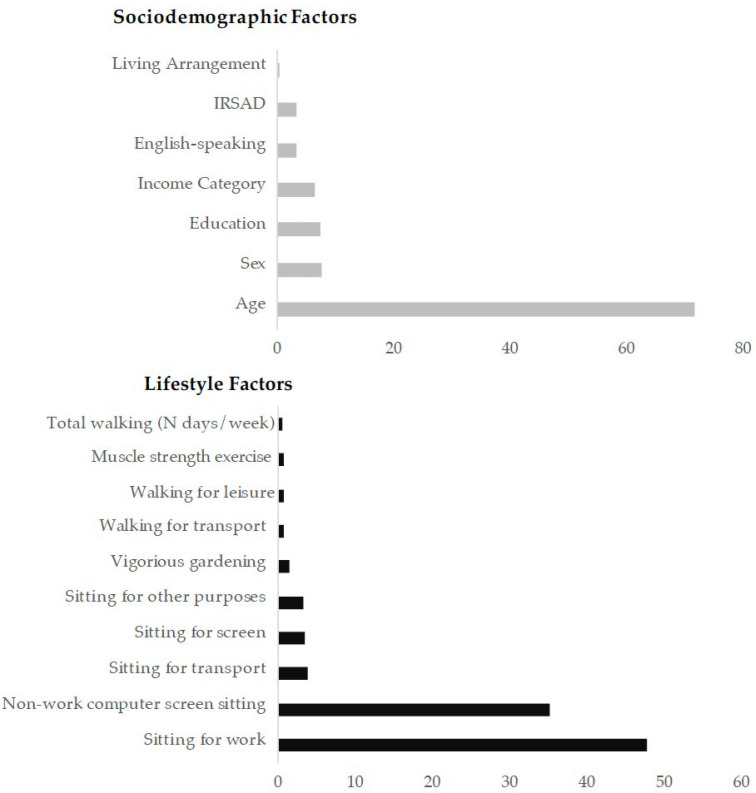
Relative contribution of the sociodemographic and lifestyle factors for predicting cognitive classes using gradient boosting machine.

The relative contribution of each sociodemographic and lifestyle factor in training the machine learning model showing the best performance is provided in Figure 2. The most important sociodemographic feature was the chronological age (72%) of the participants, followed by their sex (8%) and education (7%). The most important lifestyle feature was sedentary behaviour (sitting for work [47%] and non-work computer-screen sitting [35%]).

## 4. Discussion

This study used machine learning to predict cognitive function outcomes using sociodemographic, neighbourhood environment, and lifestyle variables in a large sample of adults. These factors are considered to be important determinants of cognitive health in ageing populations [3,30]. However, no studies thus far have examined their individual and combined predictive value in relation to individual cognitive function. Our study addressed this gap by demonstrating that sociodemographic and lifestyle factors are good predictors of cognitive function. Moreover, we highlight that the predictive value of these factors depends on the cognitive function outcome, and that the value was higher for processing speed than memory. Sociodemographic factors were the strongest predictors, with age being the most important characteristic. Lifestyle factors, including sedentary behaviour and physical activity, also significantly predicted cognitive function classes and processing speed. Neighbourhood environmental characteristics appeared to be less important when evaluated concurrently with the other predictors. A combination of all variables did not substantially improve the predictive performance.

The relationship between sociodemographic factors and cognitive decline is a well-documented finding in the literature [4,14,31,32]. Therefore, it is not surprising that sociodemographic factors such as age were one of the most important predictors of cognitive function in our study. Interestingly, in the current study, the prediction was better for processing speed compared to memory (in the regression analysis). This is likely because the speed of simple mental operations (information processing) is fundamental to cognitive impairment associated with age [33]. The SDMT task used in the current study measures information processing speed, and is, thus, more likely a better outcome measure compared to the memory task in the context of age-related cognitive decline. Processing speed is a key measure of executive function, which is linked to healthy communication within brain networks and is impaired with age [26,34]. Other sociodemographic factors, such as sex and education, also had some influence (<10%) as predictive features in training the neural network. Education is also an important sociodemographic factor associated with slowing down the ageing-related decline in cognitive function and is protective against dementia risk [4,31]. Level of education provides cognitive reserve, with magnetic resonance imaging (MRI) studies showing associations between educational attainment and hippocampal volume [31,35].

Lifestyle factors such as physical activity (leisure-time walking, resistance training, engagement in vigorous gardening) and sedentary behaviours (sitting for work, leisure, and transportation) were also good predictors of processing speed compared to memory. Furthermore, sedentary behaviours were relatively more important than physical activity for predicting cognitive function. This aligns with emerging theory that prolonged sitting impairs brain and body metabolism [36,37], a well-known risk factor for cognitive decline and all-cause dementia [38]. Sedentary behaviour and physical activity have also been found to be important determinants of cognitive function in normal ageing [1,16,18,39,40,41].

Neighbourhood environmental factors alone were found to be relatively poor predictors of cognitive health. Environmental factors such as green space and blue space can encourage physical activity and social interaction, which are important factors for maintaining good mental health [8]. However, the direct impact of environmental factors on cognition is only moderate [42,43] and can only explain between 2–10% of the variance [12]. Therefore, a lower predictive value found in the current study does not necessarily mean that the environment does not influence cognition. In fact, neighbourhood environments affect entire populations for a sustained amount of time and, hence, environmental interventions are deemed to be an important population health strategy [1].

The study also provides a comparison of the performance of various machine learning models. Therefore, some remarks regarding the relative performance of the models used in the study are warranted. The classification accuracy was high for all predictive models when using sociodemographic variables in the model, although subtle differences were evident. GBM yielded the most accurate predictions across all models (both regression and classification). Another point to note is that compared to simple LM, complex models such GBM, SVM, and ANN offered no or slight improvements in classification accuracy (AUC difference of 0.01), particularly when sociodemographic factors were included as predictors. However, GBM offered a greater improvement in classification accuracy compared to LM when only lifestyle factors were used as predictors (AUC of 0.86 vs. 0.81). These findings indicate that complex models may not necessarily improve the classification prediction ability when using small sets of sociodemographic, neighbourhood environment, and lifestyle factor variables.

A number of limitations need to be considered while interpreting the current findings. The data used in this study were cross-sectional in nature, which is a limitation. Furthermore, the lifestyle variables used in the current study were self-reported. Although this method allowed for a collection of large samples, there is a possibility of self-report bias. Another limitation is that the sample included both younger and older adults with a possibility that some adults aged 60+ may have accelerated cognitive decline. Furthermore, the study lacked information on the time participants spent within and outside the neighbourhood. This means that the environmental exposures estimated in this study may have been particularly inaccurate for those who spent a substantial amount of time outside the neighbourhood for work or other purposes. It is important to note that R-squared values for predicting memory function using sociodemographic, lifestyle, and environmental factors are rather low (<=0.2). Although this finding is consistent with previous work [44], it indicates that these variables are not explaining the large amount of variance in the memory function outcome measure. This also means that any prediction of individual memory scores using these variables alone will have high margin of error. Furthermore, we used individual learning models to predict outcome measures. Recent studies have suggested that multivariate outcome predictions using stacking algorithms may improve prediction accuracy in machine learning tasks [45]. Thus, future work will aim to use these models to improve the accuracy of our predictions. We used AUC to assess overall classification accuracy on all decision thresholds. However, if the current approach is to be used for making hard decisions on whether an individual belongs to a low or high cognitive class, other performance measures, such as AUC90, would need to be considered [45].

## 5. Conclusions

This study demonstrates that supervised learning methods hold promise for predicting cognitive function using easy-to-collect and low-cost sociodemographic, neighbourhood environmental, and lifestyle data. Leveraging a wide range of potential variables in combination can capture the unique cognitive decline risk profiles at a group or individual level. A better understanding of the variables which are predictive of decline in specific domains of cognitive function may be useful for planning interventions and informing public health policies in the increasingly ageing world.

## Figures and Tables

**Table 1:** Participant characteristics (*n* = 4141).

Characteristics	Statistics	Characteristics	Statistics
*Sociodemographic characteristics*			
Age, years, M ± SD	61.1 ± 11.4	Sex, female, %	55.2
Educational attainment, %		English-speaking background, %	89.9
Up to secondary	32.7	Household income, annual, %	
Trade, technician certificate	29.1	Up to $49,999	32.9
Associate diploma and equiv.	14.5	$50,000–$99,999	26.8
Bachelor’s degree, post-graduate diploma	23.1	$100,000 and over	28.9
		Does not know or refusal	8.8
Living arrangements, %			
Couple without children	48.2		
Couple with children	26.8		
Other	22.4		
*Neighbourhood environment attributes,* M ± SD			
Population density (persons/ha)	17.4 ± 10.0	Street intersection density	62.2 ± 32.2
Dwelling density	2.9 ± 4.2	Percentage of parkland	11.6 ± 12.5
Percentage of commercial land use	2.5 ± 6.1	Percentage of blue space	0.2 ± 1.98
Percentage of residential land use	73.6 ± 19.9	Nearest parkland (km)	0.3 ± 0.3
Nearest blue space (km)	7.9 ± 9.3	Aerial distance to trainline	3.9 ± 5.3
PM2.5	6.3 ± 1.7	Road density major roads (km)	0.9 ± 1.7
NO_2_ (ppb)	5.5 ± 2.1	Road density minor roads (km)	8.9 ± 3.7
Area-level IRSAD	6.4 ± 2.7		
*Lifestyle attributes,* M ± SD			
Vigorous gardening (times/week)	0.8 ±1.5	Muscle strength exercise	0.9 ± 2.3
Walking for transport	1.4 ± 3.5	Walking for leisure	2.4 ± 2.5
Total walking (*n* days/week)	3.1 ± 2.6		
Sitting for work	1.6 ± 2.2	Sitting for screen	1.9 ± 1.3
Sitting for transport	0.8 ± 0.8	Non-work computer sitting	0.6 ± 0.9
Sitting for other (h/day)	3.4 ± 2.4		
*Cognitive function*, M ± SD			
Memory, CVLT score	6.5 ± 2.4	Processing speed, SDMT score	49.7 ± 11.6
Missing data, %	2.3	Missing data, %	2.0

Notes. M, mean; SD, standard deviation; IRSAD, Index of Relative Socioeconomic Advantage and Disadvantage; CVLT, California Verbal Learning Test; SDMT, Symbol–Digit Modalities test; NO_2_, nitrogen dioxide; ppb, parts per billion.

**Table 2:** Coefficient of determination [*r*^2^ and 95% CI] values of association between predicted and measured processing speed (SDMT) and memory scores (CVLT). Performance of regression-based linear model (LM), support vector machine (SVM), neural network (ANN), and gradient boosting machines (GBM) are compared. Different combinations of sociodemographic (SDF), neighbourhood environment (NEF), and lifestyle (LSF) factors were used in the models.

	GBM	SVM	ANN	LM
	*r*^2^ (95% CI)	*r*^2^ (95% CI)	*r*^2^ (95% CI)	*r*^2^ (95% CI)
**SDF**				
SDMT	0.43 (0.37, 0.49)	0.43 (0.37, 0.48)	0.4 (0.33, 0.47)	0.43 (0.37, 0.48)
CVLT	0.20 (0.14, 0.27)	0.2 (0.14, 0.27)	0.18 (0.12, 0.24)	0.20 (0.14, 0.27)
**NEF**				
SDMT	0.04 (0.02, 0.07)	0.01 (0, 0.03)	0.01 (0, 0.04)	0.01 (0, 0.03)
CVLT	0.03 (0.01, 0.06)	0 (0, 0.02)	0.01 (0, 0.04)	0 (0, 0.02)
**LSF**				
SDMT	0.29 (0.23, 0.35)	0.17 (0.12, 0.23)	0.26 (0.2, 0.32)	0.17 (0.12, 0.23)
CVLT	0.10 (0.06, 0.15)	0.05 (0.01, 0.1)	0.07 (0.03, 0.11)	0.05 (0.02, 0.1)
**SDF + NEF**				
SDMT	0.43 (0.37, 0.49)	0.42 (0.36, 0.47)	0.38 (0.31, 0.45)	0.42 (0.37, 0.48)
CVLT	0.22 (0.15, 0.29)	0.2 (0.14, 0.27)	0.16 (0.1, 0.23)	0.21 (0.15, 0.28)
**SDF + LSF**				
SDMT	0.46 (0.41, 0.52)	0.43 (0.37, 0.49)	0.41 (0.35, 0.47)	0.44 (0.38, 0.49)
CVLT	0.21 (0.15, 0.28)	0.2 (0.14, 0.27)	0.16 (0.09, 0.23)	0.2 (0.14, 0.27)
**NEF + LSF**				
SDMT	0.30 (0.24, 0.36)	0.17 (0.12, 0.22)	0.24 (0.18, 0.3)	0.17 (0.12, 0.23)
CVLT	0.12 (0.07, 0.17)	0.04 (0.01, 0.09)	0.04 (0.01, 0.08)	0.05 (0.02, 0.1)
**SDF + NEF + LSF**				
SDMT	0.46 (0.41, 0.52)	0.42 (0.37, 0.48)	0.4 (0.31, 0.48)	0.43 (0.38, 0.49)
CVLT	0.23 (0.17, 0.3)	0.2 (0.14, 0.27)	0.15 (0.1, 0.22)	0.21 (0.15, 0.28)

**Table 3:** Pooled area under the curve (AUC) estimates and 95% CI for classifying cognitive function classes. Support vector machine (SVM), artificial neural network (ANN), gradient boosting machine (GBM), and linear models (LM) were used for training. Different combinations of sociodemographic (SDF), neighbourhood environment (NEF), and lifestyle (LSF) factors were used in the models.

	GBM	SVM	ANN	LM
	AUC (95% CI)	AUC (95% CI)	AUC (95% CI)	AUC (95% CI)
SDF	0.84 (0.68, 0.93)	0.84 (0.68, 0.93)	0.84 (0.67, 0.93)	0.85 (0.67, 0.94)
NEF	0.58 (0.51, 0.65)	0.53 (0.49, 0.57)	0.53 (0.45, 0.61)	0.56 (0.5, 0.61)
LSF	0.78 (0.61, 0.89)	0.62 (0.44, 0.76)	0.74 (0.61, 0.83)	0.74 (0.61, 0.85)
SDF + NEF	0.84 (0.68, 0.93)	0.84 (0.68, 0.93)	0.84 (0.68, 0.93)	0.84 (0.68, 0.93)
SDF + LSF	0.85 (0.67, 0.95)	0.84 (0.68, 0.93)	0.85 (0.68, 0.93)	0.85 (0.67, 0.94)
NEF + LSF	0.78 (0.6, 0.89)	0.64 (0.55, 0.73)	0.74 (0.62, 0.83)	0.74 (0.62, 0.83)
SDF + NEF + LSF	0.85 (0.67, 0.94)	0.84 (0.69, 0.92)	0.84 (0.68, 0.93)	0.84 (0.68, 0.93)

## Data Availability

Data that support the findings of this study are available on request under a license agreement. Written applications can be made to the AusDiab Steering Committee (Dianna.Magliano@baker.edu.au).

## Supplementary Materials

The following supporting information can be downloaded at: https://www.mdpi.com/article/10.3390/ijerph191710977/s1. Figure S1; Bayesian information criteria (BIC) plots.
