# Supplementary material for: Machine Learning for Prediction of Cognitive Health in Adults Using Sociodemographic, Neighbourhood Environmental, and Lifestyle Factors
Source: Int J Environ Res Public Health. 2022 Sep 2;19(17):10977. doi: 10.3390/ijerph191710977 (PMC9517821; doi:10.3390/ijerph191710977)
Supplement: Supplementary file 1 [file ijerph-19-10977-s001.zip › ijerph-1836705-supplementary.pdf]

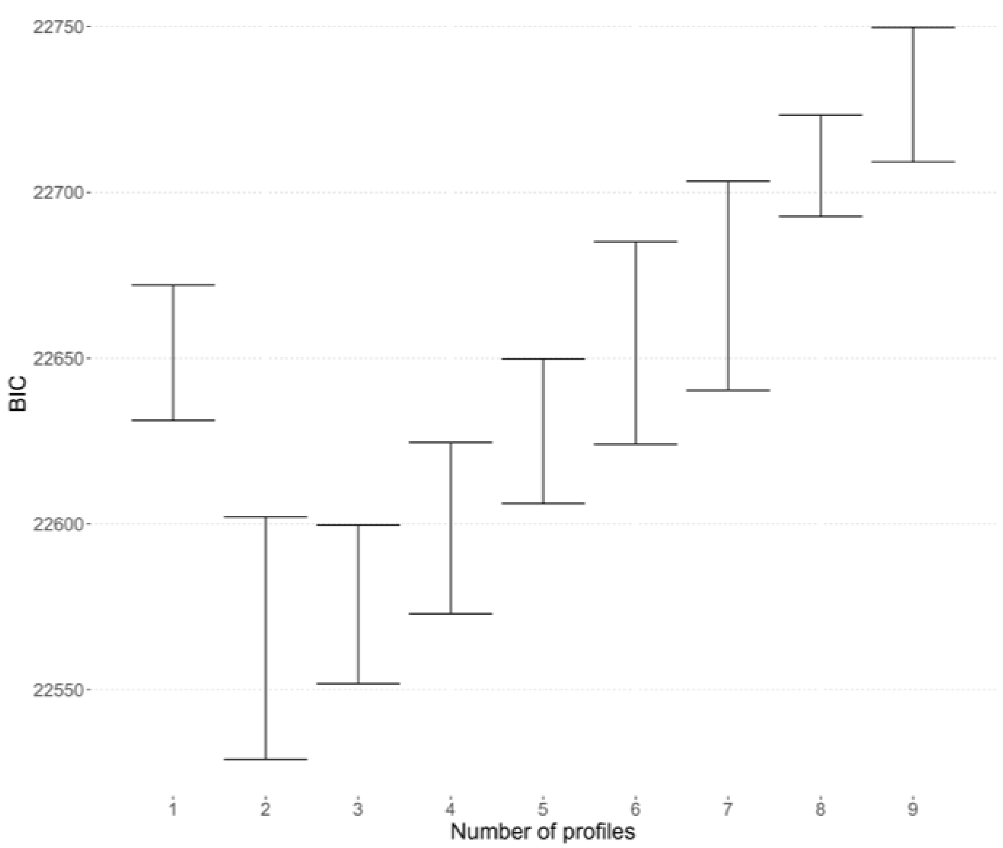

Figure S1: Bayesian information criteria (BIC) plots for the 2-class model with ellipsoidal distribution, equal shape and volume, and variable orientation (EEV). The line bar represents the range of BIC values across 10 imputations of the data. A description of the different types of models available in mclust library has been provided elsewhere (42).
